# The mediastinal width on supine anteroposterior chest radiographs

**DOI:** 10.1002/jgf2.180

**Published:** 2018-06-06

**Authors:** Junpei Komagamine

**Affiliations:** ^1^ Department of Internal Medicine National Hospital Organization Tochigi Medical Center Utsunomiya Tochigi Japan

To the Editor,

With great interest, I read the recent article reported by Funakoshi et al.^1^ The authors investigated the optimal mediastinal width on the anteroposterior chest X‐ray to differentiate nontraumatic Stanford type A acute aortic dissection (NTAD) from other diseases. They concluded that among Japanese patients with possible NTAD, a mediastinal width >87 mm showed high sensitivity, while a width >96 mm showed high specificity. However, I have several concerns about their conclusions.

First, the sensitivity of a mediastinal width >87 mm for the diagnosis of NTAD was 81% in this study. This sensitivity is not sufficiently high to rule out emergency diseases, such as NTAD. Furthermore, the sensitivity of a mediastinal width >87 mm is lower than that of a completely normal chest radiograph for the diagnosis of NTAD.^2^ Overstatements, such as “high sensitivity,” can mislead readers. Given that the diagnostic delay is critical for patients with NTAD,^3^, ^4^ more emphasis on the limitations of chest radiograph for ruling out NTAD^2^ is needed in the discussion. Second, the authors selected patients without NTAD as a control group, unlike a past study.^5^ They did not select patients who presented to the emergency department (ED) with chest pain or back pain and whose diagnosis was not NTAD. Although the difference in the control group might have a significant impact on the results, more detailed information regarding the control patients, such as the diagnosis or reason for the ED visit, is needed. Finally, there is no information on patients’ positions when chest X‐rays were performed in the Methods and Results sections in this article, although its title and abstract state “supine” anteroposterior chest radiographs. Were all chest X‐rays performed in a supine position in the ED of the authors’ hospital? This point should be clarified.

Based on the results of this study, the following conclusion is appropriate; although the sensitivity of mediastinal width on chest radiograph is not sufficiently high to rule out NTAD, a mediastinal width of >96 mm may be useful for the diagnosis of NTAD.

## CONFLICT OF INTEREST

The authors have stated explicitly that there are no conflicts of interest in connection with this article.
